# Differential long-term retention of biological disease-modifying antirheumatic drugs in patients with rheumatoid arthritis by age group from the FIRST registry

**DOI:** 10.1186/s13075-020-02233-9

**Published:** 2020-06-08

**Authors:** Akio Kawabe, Kazuhisa Nakano, Satoshi Kubo, Takeshi Asakawa, Yoshiya Tanaka

**Affiliations:** 1 The First Department of Internal Medicine, School of Medicine, University of Occupational and Environmental Health, Japan, 1-1 Iseigaoka, Yahatanishi, Kitakyushu, 807-8555 Japan; 2 Department of Information Systems Center, University of Occupational and Environmental Health, Japan, 1-1 Iseigaoka, Yahatanishi, Kitakyushu, 807-8555 Japan

**Keywords:** Rheumatoid arthritis, Biological disease-modifying antirheumatic drugs, Retention rate, Inverse probability of treatment weighting, Generalized propensity score

## Abstract

**Background:**

The effectiveness and safety of biological disease-modifying antirheumatic drugs (bDMARDs) by age group (< 65, 65–74, and ≥ 75 years) are uncertain. We examined retention rates reflecting the effectiveness and safety of bDMARDs in actual clinical practice for clarifying optimal therapeutic strategies for rheumatoid arthritis (RA) by age groups.

**Methods:**

Data of patients who were treated with tumor necrosis factor inhibitors (TNFi), abatacept (ABA), and tocilizumab (TCZ) between February 2011 and April 2017 were collected from a prospective observational registry of RA patients. A total of 1362 patients were enrolled, of which 695 were aged < 65 years, 402 were aged 65–74 years, and 265 were aged ≥ 75 years. Primary outcome was the drug retention rate in adjusted data using inverse probability of treatment weighting based on generalized propensity scores.

**Results:**

In patients aged < 65 years, 3-year retention rates of TNFi, ABA, and TCZ were 43%, 47%, and 69%, respectively (ABA versus TCZ, *p* = 0.017; TNFi versus TCZ, *p* = 0.002). In patients aged 65–74 years, 3-year retention rates of TNFi, ABA, and TCZ were 44%, 53%, and 60%, respectively (TCZ versus TNFi, *p* = 0.034). In patients aged ≥ 75 years, 3-year retention rates for TNFi, ABA, and TCZ were 38%, 63%, and 58%, respectively (ABA versus TNFi, *p* = 0.017).

**Conclusions:**

We found that the effectiveness and safety of TCZ were maximal in patients aged < 75 years and that patients aged ≥ 75 years might be suitable candidates for TCZ and ABA therapy. The use of therapeutic strategies appropriate to each age group might improve the outcomes of bDMARD therapy for RA.

## Background

Because the ratio of elderly people has markedly increased, therapeutic strategies in patients with elderly rheumatoid arthritis (RA) are emerging and important concerns to be addressed, especially in Japan which is rapidly transitioning into a super-aged society, with approximately 30% in the country being at least 65 years of age [1]. Consequently, maintaining the psychosomatic health of people aged 65–74 years and encouraging them to participate in social activities are some important issues. A new definition of true elderly people as those aged ≥ 75 years has been proposed [2]. In such a super-aged society, patients with RA are also increasingly becoming older. Thus, Japan may be a good model for developing therapeutic strategies against RA in a super-aged society that the world will face in the future.

For RA, methotrexate (MTX) and biological disease-modifying antirheumatic drugs (bDMARDs) are key options for achieving treat-to-target goals [3]. This stands true for treating elderly RA patients. However, because elderly patients exhibit age-related decreases in activities of daily living (ADL) and organ function, the incidence of adverse events (AEs) increases inevitably [4]. Accordingly, appropriate use of bDMARDs for treating RA is more important in elderly patients than in young patients. The optimal therapeutic strategy using bDMARDs for elderly and young RA patients must be reexamined. Thus, the effectiveness and safety of bDMARDs should be compared and analyzed across age groups.

Drug retention rate reflects its effectiveness and safety [5]. In elderly patients, retention rates of individual bDMARDs and of bDMARDs by age group have been investigated [6–8]. However, there has been no comparative analysis of retention rates of bDMARDs among age groups (< 65, 65–74, and ≥ 75 years). Moreover, observational studies on retention rates of individual drugs have predominantly been conducted in actual clinical practice; as such, selection bias or confounding represents a problem for comparisons between drugs.

In this study, selection bias and confounding were reduced by applying inverse probability of treatment weighting (IPTW) based on generalized propensity score [9, 10], and retention rates of bDMARDs were compared by age groups to clarify the optimal therapeutic strategies using bDMARDs.

## Methods

### Patients and study design

The FIRST registry is an observational registry designed to assess the effectiveness and safety of bDMARDs in patients with RA at our institution with a maximum follow-up period of 5 years. The risks associated with the use of bDMARDs were evaluated beforehand for each patient. The objective of this registry was to clarify the optimal therapeutic strategy using bDMARDs. Registration was initiated in March 2003; in total, 3891 patients with RA were registered until March 2020. bDMARDs included tumor necrosis factor inhibitors (TNFi) (infliximab [IFX], etanercept [ETN], adalimumab [ADA], golimumab [GLM], and certolizumab pegol [CZP]), abatacept (ABA), and tocilizumab (TCZ). This is a prospective cohort study using patient data from the FIRST registry.

In Japan, until January 2011, the maximum dose of MTX approved by the Ministry of Health, Labour and Welfare was 8 mg/week. Then, in February 2011, the maximum approved dose of MTX was increased to 16 mg/week for adults aged 20 years and above with RA. To avoid the effect of MTX restriction on the effectiveness and safety analysis, patients who received bDMARD therapy between March 2003 and January 2011 and who were aged less than 20 years were excluded from the analysis. In addition, patients whose data were missing were excluded. Patients who received bDMARD therapy between February 2011 and April 2017, who were aged 20 years and above, and who were followed up for at least 6 months were included in this study (Additional file 1: Figure S1).

According to the World Health Organization, most developed countries in the world have accepted the chronological age of 65 years for the definition of elderly [11]. The elderly population aged 65 years and above can be divided into three groups by age: young old (65–74 years), middle old (75–84 years), and oldest old (≥ 85 years) [12]. In this study, we focused on a therapeutic strategy using bDMARDs in the elderly population. Especially in the elderly population aged ≥ 75 years, frailty—an important risk factor for impaired ADL, hospitalization, and death—increases rapidly [13, 14]. This suggests that the risk of AEs is high in elderly patients aged 75 years and above. The rate of people aged ≥ 75 years has recently markedly increased in the advanced countries, and it is an emerging issue how these people can successfully improve their quality of life. Therefore, we performed subgroup analysis by dividing the elderly population into age groups of 65–74 years and ≥ 75 years, to compare the effectiveness and safety of bDMARDs among these groups.

RA was diagnosed based on the 1987 American College of Rheumatology Classification Criteria or 2010 American College of Rheumatology/European League against Rheumatism classification criteria [15–17]. The study was approved by the ethics review board of the university, and informed consent was obtained from all patients of the FIRST registry.

### bDMARD treatment

bDMARDs were prescribed to RA patients who were not adequately responsive to conventional synthetic disease-modifying antirheumatic drugs (csDMARDs) therapies. bDMARDs included both intravenous and subcutaneous agents and were administered based on the guidelines of the Japan College of Rheumatology.

### Drug retention, discontinuation, and clinical effectiveness

The primary outcome was the drug retention rate in adjusted data analysis; secondary outcomes included reasons for discontinuation in non-adjusted data analysis and clinical effectiveness in non-adjusted and adjusted data analysis. Drug retention was analyzed according to the duration of treatment until drug discontinuation at the physician’s judgment. Reasons for discontinuation were investigated using medical records and classified into discontinuation because of remission, effect insufficiency, AEs, or others (patient choice, economic, unspecified cause, and so on). Regarding discontinuation because of remission, physicians judged the state of the absence of disease activity based on composite measures such as the disease activity score using 28 joint counts (DAS28), the clinical disease activity index (CDAI), or the simple or simplified disease activity index (SDAI) [18, 19]. Moreover, regarding effect insufficiency, physicians judged increase, return, or no change in disease activity based on composite measures. AEs were further classified according to the Common Terminology Criteria for Adverse Events version 5.0 [20]. Because CDAI precisely reflects clinical response to bDMARDs, including TCZ [21], clinical effectiveness was evaluated as CDAI [22, 23].

### Statistical analysis

In the analysis of baseline characteristics, summary statistics were presented using proportions for categorical data and medians and interquartile ranges for continuous data. In the analysis of reasons for bDMARDs discontinuation, summary statistics were presented using the number of incidents and incidence. Kruskal–Wallis and chi-square tests were used to assess differences among groups. In non-adjusted data analysis, the Kaplan–Meier method was used to assess drug retention rates.

Observational studies typically involve patients who are commonly encountered in daily clinical practice; however, the study participants are subject to selection bias or confounding by indication due to the uncontrolled differences. To overcome this issue, sophisticated statistical methods are often used. Propensity score adjustment is a widely used method that attempts to control selection bias and confounding by indication in observational studies of treatment effect [24]. The propensity score matching and IPTW based on propensity score are the most popular methods applied in clinical research to reduce selection bias and confounding. However, the propensity score matching requires a number of subjects because matched patients have to be extracted from a primary study population. Also, the propensity score matching is performed for comparisons between two groups. For comparisons among multiple groups, the adjustment cannot be performed without any changes. The expanded concept of generalized propensity scores is applied to match multiple groups [9, 10]. Accordingly, in adjusted data analysis, IPTW was used based on generalized propensity scores. In this study, for covariate adjustment in the three groups of patients treated with TNFi, ABA, or TCZ, age, disease duration, gender, history of bDMARD use, MTX dose, glucocorticoid (GC) dose, tender joint count, swollen joint count, patient global assessment, evaluator global assessment, Health Assessment Questionnaire-Disability Index (HAQ-DI), C-reactive protein (CRP) level, erythrocyte sedimentation rate (ESR), and rheumatoid factor (RF) level were used as baseline covariates, and generalized propensity scores were estimated using multinomial logistic regression. Area under the curve of generalized propensity scores was > 0.7 for each bDMARD. Balance following covariate adjustment was examined, and covariate adjustment was confirmed. bDMARD retention rates were analyzed using the Kaplan–Meier method, and *p* values were calculated using the Cox proportional hazards model [10]. Since the covariates including the time-dependent covariates were adjusted using IPTW based on generalized propensity scores, the proportional hazards assumption holds.

All reported *p* values are two-sided, and the level of significance was *p* < 0.05. All analyses were performed using JMP® version 13.0.0 (SAS Institute Inc., Cary, NC, USA), SPSS® version 25 (SPSS Inc., Chicago, IL, USA), or STATA® version 14.0 (StataCorp, College Station, TX, USA).

## Results

### Patients

Additional file 1: Figure S1 shows the flow chart of patient recruitment. In total, 2139 RA patients were treated with bDMARDs between March 2003 and April 2017. We excluded 749 patients who were treated with bDMARDs between March 2003 and January 2011 and 5 patients who were < 20 years old. We also excluded 23 patients whose data were missing. Finally, 1362 patients who were treated with bDMARDs between February 2011 and April 2017 were enrolled, of which 695 were < 65 years old, 402 were 65–74 years old, and 265 were ≥ 75 years old.

### Retention rates of bDMARDs in non-adjusted data

Table 1 summarizes the characteristics of all patients and age groups. Patients aged ≥ 75 years exhibited a higher incidence of advanced-stage RA, higher ABA and GC usage, higher pre-existing lung disease prevalence, lower MTX usage, stronger inflammatory responses (e.g., CRP and ESR), higher RF levels, higher CDAI scores, and higher HAQ-DI scores than patients aged < 65 and 65–74 years.

**Table 1:** Baseline characteristics of the study population

*N*	All patients	< 65 yr	65–74 yr	≥ 75 yr	*p*
	1362	695	402	265	
Age (yr)	64 (54–72)	54 (44–60)	69 (67–72)	79 (76–81)	< 0.001
Gender (female) (%)	81.6	83.3	79.4	80.4	0.227
Disease duration (yr)	4 (1–11)	3 (0.8–9)	6 (1.4–15)	5 (1.2–15)	< 0.001
Stage (I + II) (%)	68.2	76.0	61.7	57.8	< 0.001
TNFi/ABA/TCZ (%)	59.2/25.8/15.0	65.6/17.7/16.7	57/28.4/14.7	45.7/43.4/10.9	< 0.001
Bio-naïve (%)	74.1	75.4	71.9	74	0.442
MTX use (%)	77.8	85.3	72.9	65.3	< 0.001
MTX dose (mg/w)	14 (10–16)	14 (10–16)	12(10–16)	12 (8–16)	< 0.001
GC use (%)	21.2	17.3	24.4	26.8	0.001
GC dose (mg/d)	5 (2.5–7.5)	4 (2.5–5)	5 (2.5–9.3)	5 (2.5–7.5)	0.157
CRP (mg/dL)	1.0 (0.2–3.0)	0.6 (0.1–2.2)	1.3 (0.3–3.2)	1.5 (0.4–4.2)	< 0.001
ESR (mm/h)	47 (24–75)	35 (17–62)	58 (32–80)	66 (38–85)	< 0.001
RF (IU/mL)	60 (18–167)	45 (14–130)	67 (23–188)	105 (28–215)	< 0.001
ACPA positive (%)	73.3	70.1	77.1	75.8	0.026
TJC, 0–28	7 (4–12)	7 (3–12)	7 (3–12)	8 (4–13)	0.062
SJC, 0–28	6 (3–10)	6 (3–9)	6 (3–10)	7 (4–11)	0.001
PGA, 0–100 (mm)	52 (35–72)	51 (32–71)	51 (35–70)	57 (42–76)	0.002
EGA, 0–100 (mm)	43 (29–58)	40 (26–55)	43 (30–57)	49 (34–62)	< 0.001
CDAI	24 (16–32)	23 (15–31)	24 (16–34)	27 (20–35)	< 0.001
HAQ-DI	1.3 (0.6–2.0)	1 (0.4–1.6)	1.3 (0.6–2)	1.9 (1.3–2.5)	< 0.001
Pre-existing lung disease (%)	27.4	18.4	32.3	43.4	< 0.001

Values are the median (interquartile range) unless indicated otherwise. Kruskal–Wallis and chi-square tests were used

*yr* years, *w* week, *d* day, *Stage* Steinbrocker’s stages, *TNFi* tumor necrosis factor inhibitors, *ABA* abatacept, *TCZ* tocilizumab, *Bio-naïve* biologics-naïve patients, *MTX* methotrexate, *GC* glucocorticoid, *CRP* C-reactive protein, *ESR* erythrocyte sedimentation rate, *RF* rheumatoid factor, *ACPA* anti-citrullinated peptide antibody, *TJC* tender joint count, *SJC* swollen joint count, *PGA* patient global assessment visual analogue scale, *EGA* evaluator global assessment visual analogue scale, *CDAI* clinical disease activity index, *HAQ-DI* health assessment questionnaire-disability index

Table 2 presents patient characteristics according to bDMARD usage. Additional file 1: Tables S1–3 present characteristics of each age group according to bDMARD usage. TNFi and TCZ usage decreased with age, whereas ABA usage remained comparable across age groups. TNFi-treated patients exhibited shorter disease duration, higher proportions of patients with Steinbrocker’s stage I and II RA and biologics-naïve patients, higher MTX usage, and lower pre-existing lung disease prevalence than ABA- and TCZ-treated patients. ABA-treated patients showed longer disease duration, a higher proportion of patients with Steinbrocker’s stage III and IV RA, and higher pre-existing lung disease prevalence than those treated with other bDMARDs. TCZ-treated exhibited lower MTX usage, higher GC usage, higher CRP and ESR levels, and higher CDAI scores than those treated with other bDMARDs.

**Table 2:** Baseline characteristics of patients treated with bDMARDs

*N*	TNFi	ABA	TCZ	*p*
	806	352	204	
Age (yr)	62 (51–71)	68 (60–76)	63 (54–70)	< 0.001
< 65/65–74/≥ 75 yr (%)	56.6/28.4/15	34.9/32.4/32.7	56.9/28.9/14.2	< 0.001
Gender (female) (%)	79.9	85.5	81.4	0.077
Disease duration (yr)	3 (0.8–9)	7 (2–16)	5 (1.3–12)	< 0.001
Stage (I + II) (%)	74.1	56.3	65.7	< 0.001
Bio-naïve (%)	80.1	63.6	68.1	< 0.001
MTX use (%)	87.5	66.5	58.8	< 0.001
MTX dose (mg/w)	14 (10–16)	12 (8–16)	12 (8–16)	< 0.001
GC use (%)	17.6	24.4	29.9	< 0.001
GC dose (mg/d)	4.5 (2.5–6.3)	4.5 (2.5–8)	5 (2.5–7.8)	0.672
CRP (mg/dL)	0.8 (0.2–3)	0.7 (0.1–2)	2.3 (0.7–5.3)	< 0.001
ESR (mm/h)	43 (20–73)	44 (24–71)	63 (40–85)	< 0.001
RF (IU/mL)	52 (16–151)	78 (25–199)	72 (19–161)	0.003
ACPA positive (%)	72.5	76.1	71.6	0.274
TJC, 0–28	7 (4–12)	6 (3–12)	8 (4–13)	0.008
SJC, 0–28	6 (3–10)	6 (2–9)	7 (4–11)	0.001
PGA, 0–100 (mm)	52 (35–71)	50 (33–71)	55 (38–75)	0.168
EGA, 0–100 (mm)	45 (30–60)	40 (25–52)	43 (31–59)	0.002
CDAI	24 (16–32)	22 (15–31)	26 (18–35)	0.001
HAQ-DI	1.1 (0.5–1.9)	1.4 (0.6–2.1)	1.4 (0.8–2.1)	0.004
Pre-existing lung disease (%)	22.5	39.8	25.5	< 0.001

Values are the median (interquartile range) unless indicated otherwise. Kruskal–Wallis and chi-square tests were used

*yr* years, *w* week, *d* day, *Stage* Steinbrocker’s stages, *TNFi* tumor necrosis factor inhibitors, *ABA* abatacept, *TCZ* tocilizumab, *Bio-naïve* biologics-naïve patients, *MTX* methotrexate, *GC* glucocorticoid, *CRP* C-reactive protein, *ESR* erythrocyte sedimentation rate, *RF* rheumatoid factor, *ACPA* anti-citrullinated peptide antibody, *TJC* tender joint count, *SJC* swollen joint count, *PGA* patient global assessment visual analogue scale, *EGA* evaluator global assessment visual analogue scale, *CDAI* clinical disease activity index, *HAQ-DI* health assessment questionnaire-disability index

**Fig. 1:**
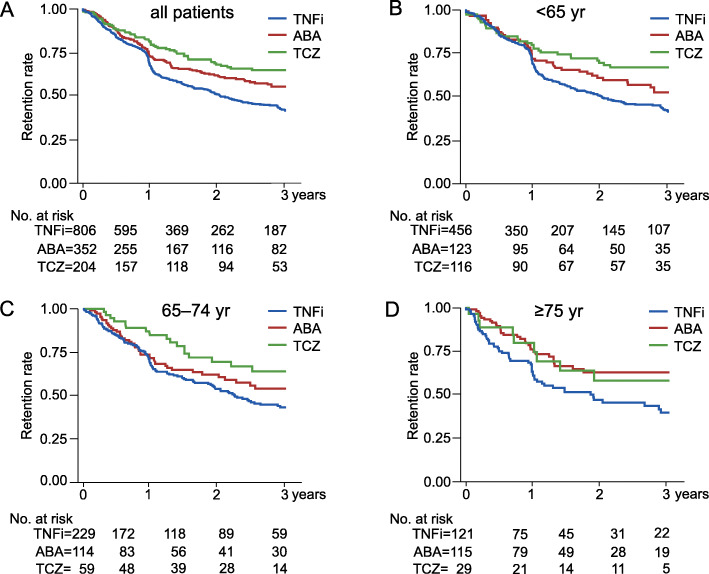
Three-year retention rates of bDMARDs by age group in non-adjusted data. Three-year retention rates of bDMARDs in all patients (**a**), in patients aged < 65 years (**b**), in patients aged 65–74 years (**c**), and in patients aged ≥ 75 years (**d**). yr = years; no. at risk = number at risk; TNFi = tumor necrosis factor inhibitors; ABA = abatacept; TCZ = tocilizumab

Three-year retention rates of bDMARDs in all patients and by age group are shown in Additional file 1: Figure S2 and Fig. 1. No significance test was performed because the data were non-adjusted. The 3-year retention rate of bDMARDs was 48.9% in all patients (Additional file 1: Figure S2A), 48.6% in patients aged < 65 years, 48.9% in patients aged 65–74 years, and 50.6% in patients aged ≥ 75 years (Additional file 1: Figure S2B). Three-year retention rates of TNFi, ABA, and TCZ were 42.6%, 55.4%, and 64.8%, respectively, in all patients (Fig. 1a). Three-year retention rates in patients aged < 65, 65–74, and ≥ 75 years were 43.1%, 43.2%, and 39.7%, respectively, for TNFi; 52.5%, 54.0%, and 62.9%, respectively, for ABA; and 67.0%, 63.9%, and 58.1%, respectively, for TCZ (Fig. 1b–d).

### Risk factors for bDMARD discontinuation in non-adjusted data

The Cox proportional hazards model was used to assess risk factors for discontinuation. Age, gender, CDAI, HAQ-DI, and bDMARDs (TNFi, ABA, or TCZ) usage were adopted for multivariate analysis. In all patients, the risk factors for drug discontinuation included age (hazard ratio [HR] = 0.992, 95% confidence interval [CI] = 0.986–0.999), female gender (HR = 0.672, 95% CI = 0.554–0.821), HAQ-DI (HR = 1.135, 95% CI = 1.013–1.272), TNFi versus ABA usage (HR = 1.301, 95% CI = 1.065–1.600), TNFi versus TCZ usage (HR = 1.919, 95% CI = 1.475–2.542), and ABA versus TCZ usage (HR = 1.474, 95% CI = 1.085–2.024) (Additional file 1: Table S4). We checked the proportional hazards assumption using the Schoenfeld residuals, and the proportional hazards assumption met the requirements of the Cox proportional hazards model. When the use of specific bDMARD was added to the Cox model, results suggested that discontinuation was related to the mechanism of action of bDMARDs (TNF blockade, IL-6 blockade, or adjustment of CD28:CD80/86 costimulatory signal) and AEs.

### Retention rates of bDMARDs in adjusted data

**Fig. 2:**
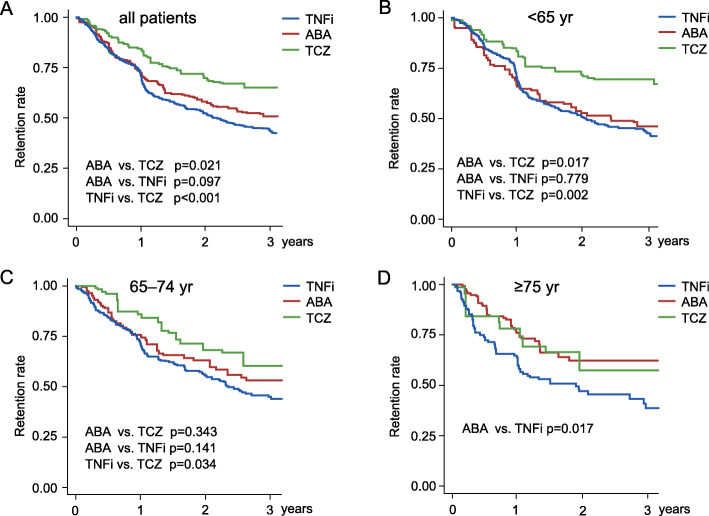
Three-year retention rates of bDMARDs in adjusted data using inverse probability of treatment weighting. Three-year retention rates of bDMARDs in all patients (**a**), in patients aged < 65 years (**b**), in patients aged 65–74 years (**c**), and in patients aged ≥ 75 years (**d**). yr = years; TNFi = tumor necrosis factor inhibitors; ABA = abatacept; TCZ = tocilizumab

Next, we focused on differences in drug retention between age-related groups. Although non-adjusted data analyses suggested great differences in drug retention rates across bDMARD type, the retention rates were affected by patient selection bias or confounding by indication. Thus, they were minimized by applying IPTW based on generalized propensity score, and the retention rate of each bDMARD was compared among the age groups (Fig. 2). Differences in baseline characteristics were adjusted by IPTW (Additional file 1: Table S5). In all patients, 3-year retention rates of TNFi, ABA, and TCZ were 43%, 51%, and 65%, respectively. The retention rate of TCZ was significantly higher than rates of ABA and TNFi (ABA versus TCZ, *p* = 0.021; TNFi versus TCZ, *p* < 0.001; Fig. 2a). In patients aged < 65 years, 3-year retention rates of TNFi, ABA, and TCZ were 43%, 47%, and 69%, respectively. The retention rate of TCZ was significantly higher than rates of ABA and TNFi (ABA versus TCZ, *p* = 0.017; TNFi versus TCZ, *p* = 0.002; Fig. 2b). In patients aged 65–74 years, 3-year retention rates of TNFi, ABA, and TCZ were 44%, 53%, and 60%, respectively. The retention rate of TCZ was significantly higher than that for TNFi (*p* = 0.034; Fig. 2c). In patients aged ≥ 75 years, 3-year retention rates for TNFi, ABA, and TCZ were 38%, 63%, and 58%, respectively. The retention rate of ABA was significantly higher than that of TNFi (*p* = 0.017; Fig. 2d). No statistical analysis was performed for TCZ usage data because of the small sample size. Overall, retention rates of TCZ were higher in patients aged < 65 and 65–74 years, and retention rates of ABA increased with age.

### Reasons for bDMARD discontinuation in non-adjusted data

Reasons for the discontinuation of each bDMARD were analyzed by age (Table 3 and Additional file 1: Table S6). In patients aged < 65 years, discontinuation because of remission was more frequent following treatment with TNFi (16.9%) and less frequent following treatment with TCZ (3.4%). The prevalence of effect insufficiency was higher following treatment with ABA (22.0%) and comparable between treatments with TNFi (14.5%) and TCZ (15.5%). AE incidence was lower following treatment with ABA (4.9%). Particularly, there was no incidence of skin and subcutaneous tissue disorders. Similarly, in patients aged 65–74 years, discontinuation because of remission was more frequent following treatment with TNFi (10.5%) and the prevalence of effect insufficiency was lower following treatment with TCZ (8.5%). AE incidence was comparable among the three drugs. Likewise, in patients aged ≥ 75 years, discontinuation because of remission was more frequent following treatment with TNFi (9.9%). The prevalence of effect insufficiency was comparable among the three drugs, and AE incidence was lower following treatment with TCZ (6.9%) and ABA (4.3%). Among AEs, the incidence of infections was lower following treatment with ABA. Overall, the rate of discontinuation because of remission was higher following treatment with TNFi, and AE incidence was lower following treatment with ABA in all age groups.

**Table 3:** Reasons for discontinuation of bDMARDs in non-adjusted data

*N*	< 65 yr			65–74 yr			≥75 yr		
	TNFi	ABA	TCZ	TNFi	ABA	TCZ	TNFi	ABA	TCZ
	456	123	116	229	114	59	121	115	29
Remission	77 (16.9)	9 (7.3)	4 (3.4)	24 (10.5)	4 (3.5)	3 (5.1)	12 (9.9)	4 (3.5)	1 (3.4)
Effect insufficiency	66 (14.5)	27 (22.0)	18 (15.5)	53 (23.1)	23 (20.2)	5 (8.5)	22 (18.2)	22 (19.1)	5 (17.2)
Adverse events	43 (9.4)	6 (4.9)	10 (8.6)	29 (12.7)	12 (10.5)	8 (13.6)	22 (18.2)	5 (4.3)	2 (6.9)
Others	44 (9.6)	8 (6.5)	2 (1.7)	15 (6.6)	3 (2.6)	1 (1.7)	4 (3.3)	5 (4.3)	1 (3.4)

Values are the number (%)

*yr* years, *TNFi* tumor necrosis factor inhibitors, *ABA* abatacept, *TCZ* tocilizumab

### Retention rates of bDMARDs in adjusted data after excluding patients who discontinued treatment because of remission

Among the reasons for bDMARD discontinuation, the rate of discontinuation because of remission was higher following treatment with TNFi in each age group. Thus, patients who discontinued treatment because of remission were excluded from the analysis. The background characteristics of the remaining patients according to age and bDMARD usage are presented in Additional file 1: Tables S7–10. Retention rates were analyzed (Additional file 1: Figure S3) after adjusting for differences in baseline characteristics using IPTW (Additional file 1: Table S11). In all patients, 3-year retention rates of TNFi, ABA, and TCZ were 48%, 55%, and 69%, respectively. The retention rate of TCZ was significantly higher than rates of ABA and TNFi (ABA versus TCZ, *p* = 0.029; TNFi versus TCZ, *p* = 0.001; Additional file 1: Figure S3A). In patients aged < 65 years, 3-year retention rates of TNFi, ABA, and TCZ were 52%, 52%, and 73%, respectively. The retention rate of TCZ was significantly higher than rates for ABA and TNFi (ABA versus TCZ, *p* = 0.025; TNFi versus TCZ, *p* = 0.011; Additional file 1: Figure S3B). In patients aged 65–74 years, 3-year retention rates of TNFi, ABA, and TCZ were 48%, 55%, and 63%, respectively. The retention rate of TCZ was significantly higher than that of TNFi (*p* = 0.048; Additional file 1: Figure S3C). In patients aged ≥ 75 years, 3-year retention rates of TNFi, ABA, and TCZ were 42%, 65%, and 63%, respectively. The retention rate of ABA was significantly higher than that of TNFi (*p* = 0.019; Additional file 1: Figure S3D). No statistical analysis was performed for TCZ data because of the small sample size. Overall, similar results to those shown in Fig. 2 were obtained even after patients who discontinued treatment because of remission were excluded.

### Changes in disease activity in non-adjusted and adjusted data

**Fig. 3:**
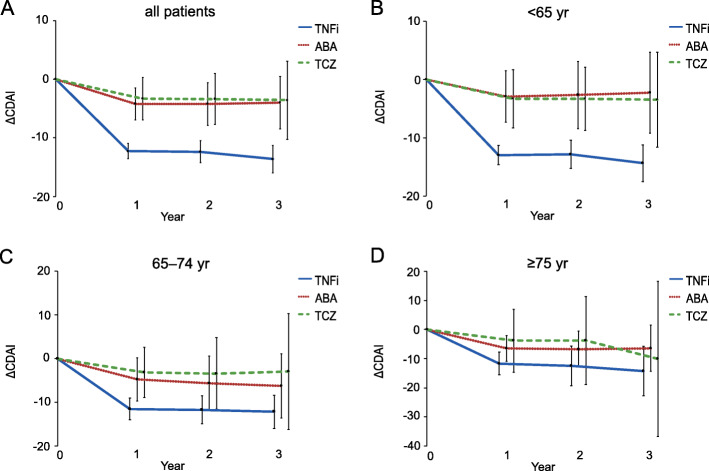
Changes in CDAI in adjusted data using inverse probability of treatment weighting. Changes in CDAI in all patients (**a**), patients aged < 65 years (**b**), patients aged 65–74 years (**c**), and patients aged ≥ 75 years (**d**). Data are presented as mean ± 95% confidence interval (error bars). CDAI = clinical disease activity index; yr = years; TNFi = tumor necrosis factor inhibitors; ABA = abatacept; TCZ = tocilizumab

Changes in CDAI were analyzed to evaluate the effects of bDMARDs. First, non-adjusted data analysis was performed to analyze CDAI at baseline and 1, 2, and 3 years (Additional file 1: Figure S4). In all patients and in each age group, CDAI tended to change in a similar manner among patients treated with TNFi, ABA, and TCZ (Additional file 1: Figure S4A, B, C, and D). Disease activity remained low in many patients who continued treatment, regardless of the bDMARD type. Furthermore, changes in CDAI from baseline to 1, 2, and 3 years were analyzed using IPTW based on generalized propensity scores (Fig. 3). In all patients, the greatest improvement in CDAI was observed with TNFi treatment, with mean differences of − 12.2 (95% CI = − 13.5 to − 10.9) at 1, − 12.3 (95% CI = − 14.2 to − 10.5) at 2, and − 13.6 (95% CI = − 15.9 to − 11.2) at 3 years (Fig. 3a). Patients aged < 65 years showed the greatest improvement in CDAI following TNFi treatment, with a mean difference of − 12.9 (95% CI = − 14.5 to − 11.3) at 1 year (Fig. 3b). Conversely, in the remaining age groups, there were no significant differences in the degree of CDAI improvement following TNFi, ABA, or TCZ treatment (Fig. 3c, d). In patients aged 65–74 and ≥ 75 years (Fig. 3c, d), differences in CDAI at 1 year were − 11.5 (95% CI = − 14.0 to − 9.0) and − 11.6 (95% CI = − 15.5 to − 7.7), respectively, for TNFi; − 4.7 (95% CI = − 9.7 to 0.2) and − 6.4 (95% CI = − 10.9 to − 2.0), respectively, for ABA; and − 3.1 (95% CI = − 8.9 to 2.6) and − 3.8 (95% CI = − 14.6 to 6.9), respectively, for TCZ. For TCZ, because of the small sample size, the 95% CIs for changes in CDAI at 2 and 3 years were wide, particularly in the older age groups, and the results for this drug should only be used as a reference.

## Discussion

In this study, after reducing selection bias and confounding by indication by applying IPTW based on generalized propensity score, the long-term retention rates of bDMARDs were examined by age group in actual clinical practice. Consequently, the following two novel points were revealed. First, in all age groups, the highest retention rate was observed for TCZ. Second, in patients aged ≥ 65 years (particularly those aged ≥ 75 years), the retention rates of ABA and TCZ were similar. Moreover, secondary assessment revealed the following two points. First, in all age groups, the lowest rate of discontinuation because of AEs was observed with ABA. Second, TNFi treatment improved disease activity more effectively and was associated with the highest rate of discontinuation because of clinical remission in all age groups.

In the analysis of “Retention rates of bDMARDs in non-adjusted data,” the retention rate was affected by patient selection bias and confounding by indication. Therefore, we used IPTW based on generalized propensity score to minimize the selection bias and confounding. The patient selection bias and confounding by indication are represented by the differences in patient background at baseline among the bDMARD groups. The results suggest that the physicians might have selected patients based on the following characteristics of each bDMARD therapy. TNFi therapy in combination with MTX is effective and may lead to discontinuation because of remission in patients with early RA [25–31]. ABA therapy is considered one of the bDMARDs that are potentially applicable to patients with infection risk and those with interstitial lung disease, according to previous studies, including the all-case postmarketing surveillance of bDMARDs in Japan [32, 33]. TCZ therapy has an advantage over other bDMARDs in the treatment of patients who cannot be treated with csDMARDs [3] and those with high CRP (as TCZ inhibits IL-6) [34]. Comorbidities are also important considerations in drug selection. Among the comorbidities, this study focused on pre-existing lung diseases. Chronic obstructive pulmonary disease and interstitial lung disease are associated with an increased risk of the development of serious infection in patients with RA receiving TNFi therapy [35]. In addition, it is considered that TNFi should be carefully selected for patients with poor respiratory reserve [33]. In contrast, it is suggested that ABA therapy has lesser adverse effects on interstitial lung disease than other bDMARDs [36].

In this study, the retention rate of TCZ was high in all age groups, particularly in patients aged < 65 years, and remained high in those aged ≥ 65 years. In a meta-analysis of studies on RA, TCZ and MTX combination was identified as the best intervention [37]. TCZ showed higher retention rates than TNFi and ABA in other studies [38, 39], which supports the results of this study. It is also reported that elderly patients with RA have higher disease activity at baseline and higher CRP level owing to age-related increases in inflammation; thus, IL-6 inhibitors may be suitable for elderly patients with RA [34, 40, 41]. The retention rates of ABA and TCZ were similar in patients aged ≥ 65 years, and this similarity is more pronounced in those aged ≥ 75 years. As a possible explanation for these findings, it is important that ABA showed the lowest discontinuation rate because of AEs in all age groups in this study and that the degree of improvement in disease activity was comparable among the drugs in patients aged ≥ 75 years. Regarding safety and age, an all-case postmarketing surveillance of bDMARDs in Japan revealed that age (≥ 65 years) contributed to severe infection onset with all biopharmaceuticals except for ABA [32, 42–45]. Long-term safety of ABA [46, 47] has been reported, and guidelines of the British Society for Rheumatology recommend ABA as the first-line treatment for patients at a risk of infection [33]. The efficacy of ABA is similar to that of TNFi [48, 49] and TCZ [50]. Regarding the effects of TNFi, there is insufficient evidence to compare retention rates between TNFi and the other bDMARDs. However, a few studies reported that the retention rate of ETN was higher than that of IFX and ADA [51, 52] and that the retention rate of IFX was lower than that of ETN and ADA [51, 53].

Elderly RA patients often present higher disease activity and greater functional impairment than young patients [34]. In recent years, immunosenescence has attracted much attention, and risk of inflammation and autoimmune diseases increases with age [54]. Immunosenescence is characterized by the lack of CD28 in T cells [54], and CD28-negative T cell count increases with age [55]. Lack of CD28 in CD4-positive T cells is associated with chronic autoimmune diseases, including RA [54]. ABA therapy reduces CD28-negative CD4-positive T cell counts, and this reduction in CD28-negative T cell count is associated with the responsiveness of RA as assessed by DAS28 based on CRP level [56, 57]. Thus, ABA may be suitable for improving immune dysregulation in elderly RA patients. However, previous studies at our institution as well as others reported that high CD28-negative CD4-positive T cell count increases the risk of decreased responsiveness to ABA therapy [58], and the responsiveness to ABA therapy improves with decreasing pretreatment CD28-negative T cell count [59]. That is, some elderly people may be at a risk of acquiring treatment resistance because of increasing CD28-negative T cell count with increasing age. These previous studies suggest that heterogeneity increases in elderly RA patients. Thus, drug selection based on both safety and treatment responsiveness is preferable.

In terms of disease activity, non-adjusted data analysis demonstrated that the degree of improvement was comparable among the three bDMARDs, although adjusted data analysis identified differences in the degree of improvement among TNFi, ABA, and TCZ. These differences suggested that patient selection bias, as indicated by differences in patient characteristics in the non-adjusted data, maximized the effect of each bDMARD in actual clinical practice. In adjusted data analysis, the improvement achieved with TNFi was greater in all age groups, and this effect was especially pronounced in patients aged < 65 years. The Remission Induction by Remicade in RA (RRR) study using IFX at our institution [26], the Humira Discontinuation without Functional and Radiographic Damage Progression following Sustained Remission (HONOR) study using ADA at our institution [27, 28], and other studies [29–31] reported that TNFi therapy may lead to discontinuation because of remission. Early-stage RA was also identified as highly reliable predictors of successful bDMARD tapering [3]. Although TNFi showed the lowest retention rate among the three bDMARDs groups, future studies are warranted to reveal characteristics of patient populations in which TNFi use is preferred and can achieve discontinuation because of remission.

This study has some limitations. First, the data were obtained from routine clinical practice, and no clear criteria were set for bDMARD discontinuation, with the decision being left at the attending physician’s discretion. Second, because IPTW based on generalized propensity score cannot exclude the effects of unknown confounding factors, this study may not have sufficiently eliminated biases compared with randomized controlled trials. Third, because IFX, ETN, ADA, GLM, and CZP were analyzed collectively as TNFi, the characteristics of each TNFi may not have been reflected. Fourth, the sample size of patients treated with TCZ was small; thus, the retention rate of TCZ among patients aged ≥ 75 years and changes in CDAI at 2 and 3 years with TCZ therapy in patients aged 65–74 and ≥ 75 years could only be used as a reference. Fifth, this study focused on the elderly population and did not perform subgroup analysis of the younger population aged less than 65 years. Sixth, history of bDMARD use may affect the retention rate of bDMARDs. However, in this study, we were unable to perform statistical analysis of subgroups, such as those treated with first-line bDMARDs and those treated with second-line or later bDMARDs, owing to the insufficient sample size. Seventh, although the emergence of new clinical evidence and new treatment options can lead to changes in the prescription practice of physicians, we could not sufficiently adjust some variables such as the calendar time of starting bDMARDs or drug approval time. Finally, this study did not include any Janus kinase inhibitors, the use of which will increase in the future.

## Conclusions

Despite the limitations, in this study, we found that the effectiveness and safety of TCZ were maximal in patients aged < 75 years and that patients aged ≥ 75 years might be suitable candidates for TCZ and ABA therapy. Furthermore, in patients aged < 65 years, TNFi improved disease activity more effectively and was associated with increased frequency of discontinuation because of remission. Finally, the use of therapeutic strategies appropriate to each age group might improve the outcomes of bDMARD therapy for RA.

## Supplementary information

**Additional file 1: Table S1.** Baseline characteristics of patients aged <65 years treated with bDMARDs. **Table S2.** Baseline characteristics of patients aged 65–74 years treated with bDMARDs. **Table S3.** Baseline characteristics of patients aged ≥75 years treated with bDMARDs. **Table S4.** Risk factors for the discontinuation of bDMARDs in all patients. **Table S5.** The generalized propensity score model in all patients. **Table S6.** Adverse events responsible for the discontinuation of bDMARDs in non-adjusted data. **Table S7.** Baseline characteristics of bDMARD groups after excluding patients who discontinued treatment because of remission. **Table S8.** Baseline characteristics of patients <65 years of age treated with bDMARDs after excluding patients who discontinued treatment because of remission. **Table S9.** Baseline characteristics of patients 65–74 years of age treated with bDMARDs after excluding patients who discontinued treatment because of remission. **Table S10.** Baseline characteristics of patients ≥75 years of age treated with bDMARDs after excluding patients who discontinued treatment because of remission. **Table S11.** Assessment of the generalized propensity score model after excluding patients who discontinued treatment because of remission. **Figure S1.** Flow chart of patient recruitment. **Figure S2.** Three-year retention rates of bDMARDs in non-adjusted data. **Figure S3.** Three-year retention rates of bDMARDs in adjusted data using inverse probability of treatment weighting after excluding patients who discontinued treatment because of remission. **Figure S4.** Changes in CDAI in non-adjusted data.

## Data Availability

The datasets used and/or analyzed during the current study are available from the corresponding author on reasonable request.

## Abbreviations

RA: Rheumatoid arthritis
MTX: Methotrexate
bDMARDs: Biological disease-modifying antirheumatic drugs
ADL: Activities of daily living
AEs: Adverse events
IPTW: Inverse probability of treatment weighting
TNFi: Tumor necrosis factor inhibitors
IFX: Infliximab
ETN: Etanercept
ADA: Adalimumab
GLM: Golimumab
CZP: Certolizumab pegol
ABA: Abatacept
TCZ: Tocilizumab
csDMARDs: Conventional synthetic disease-modifying antirheumatic drugs
DAS28: Disease activity score using 28 joint counts
CDAI: Clinical disease activity index
SDAI: Simple or simplified disease activity index
GC: Glucocorticoid
HAQ-DI: Health Assessment Questionnaire-Disability Index
CRP: C-reactive protein
ESR: Erythrocyte sedimentation rate
RF: Rheumatoid factor
HR: Hazard ratio
CI: Confidence interval
