# Supplementary material for: Differential long-term retention of biological disease-modifying antirheumatic drugs in patients with rheumatoid arthritis by age group from the FIRST registry
Source: Arthritis Res Ther. 2020 Jun 8;22:136. doi: 10.1186/s13075-020-02233-9 (PMC7282084; doi:10.1186/s13075-020-02233-9)
Supplement: Supplementary file 1 — Additional file 1: Table S1. Baseline characteristics of patients aged <65 years treated with bDMARDs. Table S2. Baseline characteristics of patients aged 65–74 years treated with bDMARDs. Table S3. Baseline characteristics of patients aged ≥75 years treated with bDMARDs. Table S4. Risk factors for the discontinuation of bDMARDs in all patients. Table S5. The generalized propensity score model in all patients. Table S6. Adverse events responsible for the discontinuation of bDMARDs in non-adjusted data. Table S7. Baseline characteristics of bDMARD groups after excluding patients who discontinued treatment because of remission. Table S8. Baseline characteristics of patients <65 years of age treated with bDMARDs after excluding patients who discontinued treatment because of remission. Table S9. Baseline characteristics of patients 65–74 years of age treated with bDMARDs after excluding patients who discontinued treatment because of remission. Table S10. Baseline characteristics of patients ≥75 years of age treated with bDMARDs after excluding patients who discontinued treatment because of remission. Table S11. Assessment of the generalized propensity score model after excluding patients who discontinued treatment because of remission. Figure S1. Flow chart of patient recruitment. Figure S2. Three-year retention rates of bDMARDs in non-adjusted data. Figure S3. Three-year retention rates of bDMARDs in adjusted data using inverse probability of treatment weighting after excluding patients who discontinued treatment because of remission. Figure S4. Changes in CDAI in non-adjusted data. [file 13075_2020_2233_MOESM1_ESM.docx]

**Table S1.** Baseline characteristics of patients aged <65 years treated with bDMARDs

| N | TNFi  456 | ABA  123 | TCZ  116 | *p* |
| --- | --- | --- | --- | --- |
| Age (yr) | 53 (42–59) | 56 (45–61) | 57 (49–61) | 0.003 |
| Gender (female) (%) | 81.6 | 88.6 | 84.5 | 0.166 |
| Disease duration (yr) | 2.1 (0.7–7) | 5.4 (1.3–11) | 4 (0.9–12) | <0.001 |
| Stage (I + II) (%) | 81.6 | 64.2 | 66.3 | <0.001 |
| Bio-naïve (%) | 81.4 | 61.0 | 67.2 | <0.001 |
| MTX use (%) | 91.4 | 80.5 | 66.4 | <0.001 |
| MTX dose (mg/w) | 15 (12–16) | 12 (10–16) | 14 (10–16) | 0.005 |
| GC use (%) | 12.9 | 23.6 | 27.6 | <0.001 |
| GC dose (mg/d) | 4 (2.5–5) | 3 (2–5) | 4.8 (2.1–7.5) | 0.717 |
| CRP (mg/dL) | 0.5 (0.1–1.9) | 0.4 (0.1–1.3) | 1.9 (0.3–5.2) | <0.001 |
| ESR (mm/h) | 32 (16–60) | 31 (14–52) | 53 (32–79) | <0.001 |
| RF (IU/mL) | 43 (14–128) | 37 (14–135) | 67 (15–136) | 0.452 |
| ACPA positive (%) | 71.3 | 68.3 | 67.2 | 0.739 |
| TJC, 0–28 | 6 (4–11) | 6 (2–11) | 9 (4–14) | 0.010 |
| SJC, 0–28 | 5 (3–9) | 5 (2–8) | 6 (4–10) | <0.001 |
| PGA VAS, 0–100 (mm) | 51 (31–70) | 50 (25–70) | 60 (45–75) | 0.014 |
| EGA VAS, 0–100 (mm) | 40 (27–55) | 34 (21–50) | 45 (33–60) | 0.001 |
| CDAI | 23 (15–31) | 20 (12–29) | 26 (19–34) | <0.001 |
| HAQ-DI | 0.9 (0.4–1.5) | 1 (0.4–1.6) | 1.3 (0.9–2) | <0.001 |
| Pre-existing lung disease (%) | 14.5 | 31.7 | 19.8 | <0.001 |

Values are the median (interquartile range) unless indicated otherwise. Kruskal–Wallis and chi-square tests were used. yr = years; w = week; d = day; Stage = Steinbrocker’s stages; TNFi = tumor necrosis factor inhibitors; ABA = abatacept; TCZ = tocilizumab; Bio-naïve = biologics-naïve patients; MTX = methotrexate; GC = glucocorticoid; CRP = C-reactive protein; ESR = erythrocyte sedimentation rate; RF = rheumatoid factor; ACPA = anti-citrullinated peptide antibody; TJC = tender joint count; SJC = swollen joint count; PGA = patient global assessment visual analogue scale; EGA = evaluator global assessment visual analogue scale; CDAI = clinical disease activity index; HAQ-DI = health assessment questionnaire-disability index.

**Table S2.** Baseline characteristics of patients aged 65–74 years treated with bDMARDs

| N | TNFi  229 | ABA  114 | TCZ  59 | *p* |
| --- | --- | --- | --- | --- |
| Age (yr) | 70 (67–72) | 69 (67–72) | 69 (67–71) | 0.761 |
| Gender (female) (%) | 77.3 | 85.1 | 76.3 | 0.200 |
| Disease duration (yr) | 5 (1–13) | 8.6 (2–18) | 5.7 (1.8–14) | 0.037 |
| Stage (I + II) (%) | 65.1 | 53.5 | 64.4 | 0.059 |
| Bio-naïve (%) | 76.9 | 63.2 | 69.5 | 0.026 |
| MTX use (%) | 87.8 | 54.4 | 50.8 | <0.001 |
| MTX dose (mg/w) | 12 (10–16) | 12 (8–16) | 12 (9.5–16) | 0.286 |
| GC use (%) | 22.3 | 24.6 | 32.2 | 0.285 |
| GC dose (mg/d) | 4.5 (2.5–5) | 5 (3.6–10) | 4.0 (2.5–10) | 0.393 |
| CRP (mg/dL) | 1.2 (0.3–3.4) | 0.9 (0.1–2.2) | 2.4 (1.2–4.7) | <0.001 |
| ESR (mm/h) | 56 (32–77) | 54 (29–77) | 71 (56–90) | <0.001 |
| RF (IU/mL) | 64 (21–185) | 75 (27–190) | 93 (25–204) | 0.692 |
| ACPA positive (%) | 76.9 | 77.2 | 78 | 0.783 |
| TJC, 0–28 | 7 (3–13) | 6 (2–12) | 7 (4–12) | 0.112 |
| SJC, 0–28 | 6 (3–11) | 6 (3–10) | 8 (4–10) | 0.348 |
| PGA VAS, 0–100 (mm) | 53 (40–72) | 51 (34–70) | 48 (29–68) | 0.214 |
| EGA VAS, 0–100 (mm) | 46 (32–61) | 39 (25–50) | 40 (28–53) | 0.002 |
| CDAI | 25 (17–35) | 22 (15–31) | 24 (17–34) | 0.109 |
| HAQ-DI | 1.3 (0.8–2.1) | 1.4 (0.6–2) | 1.1 (0.5–2.1) | 0.756 |
| Pre-existing lung disease (%) | 27.1 | 44.7 | 28.8 | 0.004 |

Values are the median (interquartile range) unless indicated otherwise. Kruskal–Wallis and chi-square tests were used. yr = years; w = week; d = day; Stage = Steinbrocker’s stages; TNFi = tumor necrosis factor inhibitors; ABA = abatacept; TCZ = tocilizumab; Bio-naïve = biologics-naïve patients; MTX = methotrexate; GC = glucocorticoid; CRP = C-reactive protein; ESR = erythrocyte sedimentation rate; RF = rheumatoid factor; ACPA = anti-citrullinated peptide antibody; TJC = tender joint count; SJC = swollen joint count; PGA = patient global assessment visual analogue scale; EGA = evaluator global assessment visual analogue scale; CDAI = clinical disease activity index; HAQ-DI = health assessment questionnaire-disability index.

**Table S3.** Baseline characteristics of patients aged ≥75 years treated with bDMARDs

| N | TNFi  121 | ABA  115 | TCZ  29 | *p* |
| --- | --- | --- | --- | --- |
| Age (yr) | 79 (76–81) | 79 (76–82) | 78 (76–81) | 0.632 |
| Gender (female) (%) | 78.5 | 82.6 | 79.3 | 0.722 |
| Disease duration (yr) | 2.7 (0.7–11) | 9 (2.7–20) | 5 (1.3–19) | <0.001 |
| Stage (I + II) (%) | 62.8 | 50.4 | 65.5 | 0.028 |
| Bio-naïve (%) | 81.8 | 67 | 69 | 0.028 |
| MTX use (%) | 71.9 | 63.5 | 44.8 | 0.020 |
| MTX dose (mg/w) | 12 (8–16) | 12 (6.5–14) | 10 (6.5–16) | 0.034 |
| GC use (%) | 26.4 | 25.2 | 34.5 | 0.598 |
| GC dose (mg/d) | 5 (3–10) | 3 (2.3–5) | 5 (4.8–11) | 0.067 |
| CRP (mg/dL) | 1.6 (0.4–4.4) | 1.2 (0.2–3) | 4.6 (1.6–7.7) | <0.001 |
| ESR (mm/h) | 66 (37–84) | 62 (33–83) | 83 (60–99) | 0.003 |
| RF (IU/mL) | 72 (14–172) | 136 (50–283) | 107 (20–201) | 0.001 |
| ACPA positive (%) | 68.6 | 83.5 | 75.9 | 0.034 |
| TJC, 0–28 | 8 (5–13) | 8 (4–12) | 9 (5–14) | 0.588 |
| SJC, 0–28 | 7 (4–11) | 6 (4–10) | 10 (4–15) | 0.041 |
| PGA VAS, 0–100 (mm) | 58 (45–76) | 54 (41–75) | 75 (42–83) | 0.110 |
| EGA VAS, 0–100 (mm) | 50 (37–67) | 46 (33–60) | 46 (32–55) | 0.106 |
| CDAI | 28 (20–35) | 25 (19–32) | 28 (22–41) | 0.109 |
| HAQ-DI | 1.9 (1.3–2.5) | 1.9 (1.3–2.5) | 1.9 (1.4–2.6) | 0.930 |
| Pre-existing lung disease (%) | 43.8 | 43.5 | 41.4 | 0.972 |

Values are the median (interquartile range) unless indicated otherwise. Kruskal–Wallis and chi-square tests were used. yr = years; w = week; d = day; Stage = Steinbrocker’s stages; TNFi = tumor necrosis factor inhibitors; ABA = abatacept; TCZ = tocilizumab; Bio-naïve = biologics-naïve patients; MTX = methotrexate; GC = glucocorticoid; CRP = C-reactive protein; ESR = erythrocyte sedimentation rate; RF = rheumatoid factor; ACPA = anti-citrullinated peptide antibody; TJC = tender joint count; SJC = swollen joint count; PGA = patient global assessment visual analogue scale; EGA = evaluator global assessment visual analogue scale; CDAI = clinical disease activity index; HAQ-DI = health assessment questionnaire-disability index.

**Table S4.** Risk factors for the discontinuation of bDMARDs in all patients

|  | Univariate | Multivariate |  |
| --- | --- | --- | --- |
|  | *p* | Hazard ratio (95% CI) | *p* |
| Age | 0.761 | **0.992 (0.986–0.999)** | **0.025** |
| Female | <0.001 | **0.672 (0.554–0.821)** | **<0.001** |
| Disease duration | 0.199 |  |  |
| Stage I + II | 0.191 |  |  |
| Bio-naïve | 0.117 |  |  |
| MTX use | 0.310 |  |  |
| GC use | 0.910 |  |  |
| CRP | 0.341 |  |  |
| RF positive | 0.609 |  |  |
| ACPA positive | 0.658 |  |  |
| CDAI | 0.068 | 1.004 (0.997–1.011) | 0.189 |
| HAQ-DI | 0.095 | **1.135 (1.013–1.272)** | **0.028** |
| Pre-existing lung disease | 0.264 |  |  |
| bDMARDs | <0.001 |  |  |
| TNFi vs. ABA |  | **1.301 (1.065–1.600)** | **0.009** |
| TNFi vs. TCZ |  | **1.919 (1.475–2.542)** | **<0.001** |
| ABA vs. TCZ |  | **1.474 (1.085–2.024)** | **0.012** |

The Cox proportional hazards model was used to assess risk factors for discontinuation. Age and variables with *p* < 0.1 in univariate analysis (the log-rank test) were adopted for multivariate analysis. In univariate analysis, median was used to split continuous variables into groups. 95% CI = 95% confidence interval; Stage = Steinbrocker’s stages; Bio-naïve = biologics-naïve patients; MTX = methotrexate; GC = glucocorticoid; CRP = C-reactive protein; RF = rheumatoid factor; ACPA = anti-citrullinated peptide antibody; CDAI = clinical disease activity index; HAQ-DI = health assessment questionnaire-disability index; bDMARDs = biological disease-modifying antirheumatic drugs; TNFi = tumor necrosis factor inhibitors; ABA = abatacept; TCZ = tocilizumab.

**Table S5.** The generalized propensity score model in all patients

| AUC | TNFi  0.718 | | ABA  0.725 | | TCZ  0.733 | |
| --- | --- | --- | --- | --- | --- | --- |
|  | *p* | | *p* | | *p* | |
| IPTW | before | after | before | after | before | after |
| covariates |  |  |  |  |  |  |
| Age | <0.001 | 0.601 | <0.001 | 0.770 | 0.847 | 0.775 |
| Disease duration | <0.001 | 0.910 | <0.001 | 0.861 | 0.276 | 0.723 |
| Gender | 0.055 | 0.345 | 0.026 | 0.818 | 0.936 | 0.858 |
| History of bDMARD use | <0.001 | 0.281 | <0.001 | 0.798 | 0.035 | 0.802 |
| MTX dose | <0.001 | 0.788 | <0.001 | 0.892 | <0.001 | 0.704 |
| GC dose | <0.001 | 0.692 | 0.194 | 0.682 | <0.001 | 0.972 |
| TJC, 0–28 | 0.971 | 0.472 | 0.051 | 0.784 | 0.019 | 0.890 |
| SJC, 0–28 | 0.353 | 0.556 | 0.001 | 0.857 | 0.007 | 0.799 |
| PGA | 0.649 | 0.742 | 0.243 | 0.880 | 0.039 | 0.767 |
| EGA | 0.009 | 0.879 | <0.001 | 0.810 | 0.377 | 0.719 |
| HAQ-DI | 0.001 | 0.672 | 0.031 | 0.719 | 0.074 | 0.747 |
| CRP | 0.757 | 0.894 | <0.001 | 0.724 | <0.001 | 0.565 |
| ESR | <0.001 | 0.787 | 0.175 | 0.719 | <0.001 | 0.580 |
| RF | 0.220 | 0.983 | 0.046 | 0.923 | 0.451 | 0.964 |

Mann–Whitney U test, *t*-test, or tests for two independent proportions were used. AUC = area under the curve; IPTW = inverse probability of treatment weighting; TNFi = tumor necrosis factor inhibitors; ABA = abatacept; TCZ = tocilizumab; bDMARD = biological disease-modifying antirheumatic drug; MTX = methotrexate; GC = glucocorticoid; TJC = tender joint count; SJC = swollen joint count; PGA = patient global assessment visual analogue scale; EGA = evaluator global assessment visual analogue scale; HAQ-DI = health assessment questionnaire-disability index; CRP = C-reactive protein; ESR = erythrocyte sedimentation rate; RF = rheumatoid factor.

**Table S6.** Adverse events responsible for the discontinuation of bDMARDs in non-adjusted data

|  | <65 yr | | | 65–74 yr | | | ≥75 yr | | |
| --- | --- | --- | --- | --- | --- | --- | --- | --- | --- |
| N | TNFi  456 | ABA  123 | TCZ  116 | TNFi  229 | ABA  114 | TCZ  59 | TNFi  121 | ABA  115 | TCZ  29 |
| Infections and infestations | 4 (0.9) | 1 (0.8) | 3 (2.6) | 3 (1.3) | 2 (1.8) | 3 (5.1) | 6 (5.0) | 1 (0.9) | 1 (3.4) |
| General disorders and administration site conditions | 14 (3.1) | 1 (0.8) | 1 (0.9) | 7 (3.1) | 5 (4.4) | 1 (1.7) | 4 (3.3) | 1 (0.9) | 0 |
| Skin and subcutaneous tissue disorders | 6 (1.3) | 0 | 4 (3.4) | 7 (3.1) | 2 (1.8) | 3 (5.1) | 4 (3.3) | 0 | 1 (3.4) |
| Nervous system disorders | 1 (0.2) | 0 | 0 | 1 (0.4) | 1 (0.9) | 0 | 3 (2.5) | 2 (1.7) | 0 |
| Neoplasms benign, malignant and unspecified | 8 (1.8) | 1 (0.8) | 0 | 6 (2.6) | 0 | 1 (1.7) | 2 (1.7) | 0 | 0 |
| Immune system disorders | 2 (0.4) | 0 | 0 | 1 (0.4) | 0 | 0 | 1 (0.8) | 0 | 0 |
| Injury, poisoning and procedural complications | 1 (0.2) | 0 | 0 | 2 (0.9) | 0 | 0 | 1 (0.8) | 0 | 0 |
| Respiratory, thoracic and mediastinal disorders | 3 (0.7) | 1 (0.8) | 1 (0.9) | 5 (2.2) | 1 (0.9) | 0 | 1 (0.8) | 0 | 0 |
| Musculoskeletal and connective tissue disorders | 0 | 0 | 0 | 1 (0.4) | 0 | 0 | 0 | 0 | 0 |
| Investigations | 2 (0.4) | 0 | 1 (0.9) | 2 (0.9) | 1 (0.9) | 0 | 0 | 0 | 0 |
| Blood and lymphatic system disorders | 1 (0.2) | 0 | 0 | 0 | 0 | 0 | 0 | 0 | 0 |
| Vascular disorders and Cardiac disorders | 1 (0.2) | 0 | 0 | 1 (0.4) | 0 | 0 | 0 | 0 | 0 |
| Gastrointestinal disorders | 0 | 2 (1.6) | 1 (0.9) | 3 (1.3) | 0 | 0 | 0 | 1 (0.9) | 0 |
| Renal and urinary disorders | 0 | 0 | 0 | 0 | 1 (0.9) | 0 | 0 | 1 (0.9) | 0 |

Values are the number (%). yr = years; TNFi = tumor necrosis factor inhibitors; ABA = abatacept; TCZ = tocilizumab.

**Table S7.** Baseline characteristics of bDMARD groups after excluding patients who discontinued treatment because of remission

| N | TNFi  693 | ABA  335 | TCZ  196 | *p* |
| --- | --- | --- | --- | --- |
| Age (yr) | 63 (51–71) | 69 (61–76) | 63 (54–70) | <0.001 |
| <65/65–74/≥75 yr (%) | 54.7/29.6/15.7 | 34/32.8/33.1 | 57.1/28.6/14.3 | <0.001 |
| Gender (female) (%) | 80.5 | 85.7 | 81.6 | 0.128 |
| Disease duration (yr) | 3.4 (0.9–10) | 7 (2–16) | 4.9 (1.3–12) | <0.001 |
| Stage (I + II) (%) | 72.1 | 56.7 | 65.3 | <0.001 |
| Bio-naïve (%) | 77.9 | 64.5 | 66.8 | <0.001 |
| MTX use (%) | 85.9 | 66 | 57.7 | <0.001 |
| MTX dose (mg/w) | 14 (10–16) | 12 (8–16) | 12 (10–16) | <0.001 |
| GC use (%) | 19 | 25.4 | 30.6 | 0.001 |
| GC dose (mg/d) | 4.8 (2.5–5.8) | 5 (2.5–8) | 5 (2.5–7.9) | 0.620 |
| CRP (mg/dL) | 0.8 (0.2–3.2) | 0.7 (0.1–2.1) | 2.3 (0.7–5.3) | <0.001 |
| ESR (mm/h) | 44 (22–73) | 46 (24–73) | 63 (42–85) | <0.001 |
| RF (IU/mL) | 56 (16–157) | 80 (26–200) | 71 (18–166) | 0.005 |
| ACPA positive (%) | 73.2 | 76.1 | 70.9 | 0.361 |
| TJC, 0–28 | 7 (4–12) | 6 (3–12) | 9 (5–13) | 0.015 |
| SJC, 0–28 | 6 (3–10) | 6 (3–9) | 7 (4–11) | 0.001 |
| PGA, 0–100 (mm) | 53 (36–73) | 51 (36–72) | 56 (38–75) | 0.412 |
| EGA, 0–100 (mm) | 45 (30–60) | 40 (26–52) | 43 (32–59) | 0.003 |
| CDAI | 24 (17–34) | 23 (15–31) | 26 (19–35) | 0.003 |
| HAQ-DI | 1.3 (0.6–2) | 1.4 (0.6–2.1) | 1.4 (0.8–2.1) | 0.047 |
| Pre-existing lung disease (%) | 24.2 | 40.3 | 26.5 | <0.001 |

Values are the median (interquartile range) unless indicated otherwise. Kruskal–Wallis and chi-square tests were used. yr = years; w = week; d = day; Stage = Steinbrocker’s stages; TNFi = tumor necrosis factor inhibitors; ABA = abatacept; TCZ = tocilizumab; Bio-naïve = biologics-naïve patients; MTX = methotrexate; GC = glucocorticoid; CRP = C-reactive protein; ESR = erythrocyte sedimentation rate; RF = rheumatoid factor; ACPA = anti-citrullinated peptide antibody; TJC = tender joint count; SJC = swollen joint count; PGA = patient global assessment visual analogue scale; EGA = evaluator global assessment visual analogue scale; CDAI = clinical disease activity index; HAQ-DI = health assessment questionnaire-disability index.

**Table S8.** Baseline characteristics of patients <65 years of age treated with bDMARDs after excluding patients who discontinued treatment because of remission

| N | TNFi  379 | ABA  114 | TCZ  112 | *p* |
| --- | --- | --- | --- | --- |
| Age (yr) | 53 (42–59) | 56 (46–61) | 57 (50–61) | <0.001 |
| Gender (female) (%) | 81.8 | 89.5 | 83.9 | 0.151 |
| Disease duration (yr) | 2.8 (0.8–8) | 5 (1.2–11) | 3.7 (0.9–12) | 0.006 |
| Stage (I + II) (%) | 80.2 | 65.8 | 66.1 | 0.004 |
| Bio-naïve (%) | 79.4 | 63.2 | 66.1 | <0.001 |
| MTX use (%) | 90.2 | 79.8 | 65.2 | <0.001 |
| MTX dose (mg/w) | 14 (10–16) | 12 (8–16) | 12 (8–16) | 0.011 |
| GC use (%) | 15 | 25.4 | 27.7 | 0.002 |
| GC dose (mg/d) | 4 (2.3–5) | 3 (2–5) | 5 (2.5–7.5) | 0.611 |
| CRP (mg/dL) | 0.6 (0.1–2.2) | 0.4 (0.1–1.4) | 2 (0.4–5.2) | <0.001 |
| ESR (mm/h) | 32 (16–62) | 32 (14–52) | 53 (33–79) | <0.001 |
| RF (IU/mL) | 45 (14–130) | 39 (16–156) | 64 (15–136) | 0.715 |
| ACPA positive (%) | 72.7 | 68.8 | 68.8 | 0.589 |
| TJC, 0–28 | 7 (4–12) | 6 (3–12) | 9 (4–14) | 0.042 |
| SJC, 0–28 | 6 (3–10) | 5 (2–8) | 7 (4–10) | 0.002 |
| PGA VAS, 0–100 (mm) | 52 (32–72) | 50 (26–70) | 60 (45–75) | 0.066 |
| EGA VAS, 0–100 (mm) | 43 (28–58) | 36 (22–50) | 45 (33–60) | 0.004 |
| CDAI | 23 (16–32) | 20 (13–29) | 26 (19–34) | 0.002 |
| HAQ-DI | 1 (0.5–1.6) | 1.1 (0.4–1.6) | 1.3 (0.9–2) | 0.001 |
| Pre-existing lung disease (%) | 15.6 | 32.5 | 20.5 | <0.001 |

Values are the median (interquartile range) unless indicated otherwise. Kruskal–Wallis and chi-square tests were used. yr = years; w = week; d = day; Stage = Steinbrocker’s stages; TNFi = tumor necrosis factor inhibitors; ABA = abatacept; TCZ = tocilizumab; Bio-naïve = biologics-naïve patients; MTX = methotrexate; GC = glucocorticoid; CRP = C-reactive protein; ESR = erythrocyte sedimentation rate; RF = rheumatoid factor; ACPA = anti-citrullinated peptide antibody; TJC = tender joint count; SJC = swollen joint count; PGA = patient global assessment visual analogue scale; EGA = evaluator global assessment visual analogue scale; CDAI = clinical disease activity index; HAQ-DI = health assessment questionnaire-disability index.

**Table S9.** Baseline characteristics of patients 65–74 years of age treated with bDMARDs after excluding patients who discontinued treatment because of remission

| N | TNFi  205 | ABA  110 | TCZ  56 | *p* |
| --- | --- | --- | --- | --- |
| Age (yr) | 70 (67–72) | 69 (67–72) | 69 (67–71) | 0.794 |
| Gender (female) (%) | 78.5 | 84.5 | 78.6 | 0.414 |
| Disease duration (yr) | 5.8 (1.1–14) | 8.6 (2–18) | 6.3 (1.8–14) | 0.201 |
| Stage (I + II) (%) | 63.4 | 52.7 | 64.3 | 0.201 |
| Bio-naïve (%) | 74.1 | 63.6 | 67.9 | 0.140 |
| MTX use (%) | 86.3 | 53.6 | 50 | <0.001 |
| MTX dose (mg/w) | 12 (10–16) | 12 (8–16) | 12 (10–16) | 0.312 |
| GC use (%) | 22.4 | 25.5 | 33.9 | 0.212 |
| GC dose (mg/d) | 4.8 (2.5–5) | 5 (3.6–10) | 4 (2.5–10) | 0.370 |
| CRP (mg/dL) | 1.2 (0.3–3.7) | 0.9 (0.1–2.2) | 2.3 (1.1–4.7) | <0.001 |
| ESR (mm/h) | 56 (32–79) | 54 (29–77) | 72 (56–90) | 0.001 |
| RF (IU/mL) | 66 (22–192) | 76 (28–197) | 90 (25–218) | 0.836 |
| ACPA positive (%) | 78 | 80.2 | 78.2 | 0.905 |
| TJC, 0–28 | 8 (4–13) | 6 (2–12) | 8 (4–12) | 0.138 |
| SJC, 0–28 | 7 (4–11) | 6 (3–10) | 8 (4–10) | 0.365 |
| PGA VAS, 0–100 (mm) | 53 (41–72) | 51 (37–70) | 47 (29–68) | 0.147 |
| EGA VAS, 0–100 (mm) | 47 (33–62) | 40 (27–50) | 40 (28–53) | 0.002 |
| CDAI | 26 (18–35) | 22 (15–32) | 24 (17–34) | 0.103 |
| HAQ-DI | 1.4 (0.8–2.1) | 1.4 (0.6–2) | 1.1 (0.5–2.2) | 0.567 |
| Pre-existing lung disease (%) | 27.8 | 45.5 | 30.4 | 0.006 |

Values are the median (interquartile range) unless indicated otherwise. Kruskal–Wallis and chi-square tests were used. yr = years; w = week; d = day; Stage = Steinbrocker’s stages; TNFi = tumor necrosis factor inhibitors; ABA = abatacept; TCZ = tocilizumab; Bio-naïve = biologics-naïve patients; MTX = methotrexate; GC = glucocorticoid; CRP = C-reactive protein; ESR = erythrocyte sedimentation rate; RF = rheumatoid factor; ACPA = anti-citrullinated peptide antibody; TJC = tender joint count; SJC = swollen joint count; PGA = patient global assessment visual analogue scale; EGA = evaluator global assessment visual analogue scale; CDAI = clinical disease activity index; HAQ-DI = health assessment questionnaire-disability index.

**Table S10.** Baseline characteristics of patients ≥75 years of age treated with bDMARDs after excluding patients who discontinued treatment because of remission

| N | TNFi  109 | ABA  111 | TCZ  28 | *p* |
| --- | --- | --- | --- | --- |
| Age (yr) | 79 (76–82) | 79 (76–82) | 78 (76–81) | 0.649 |
| Gender (female) (%) | 79.8 | 82.9 | 78.6 | 0.793 |
| Disease duration (yr) | 4 (0.7–13) | 9.1 (2.7–21) | 4.5 (1.3–19) | <0.001 |
| Stage (I + II) (%) | 60.6 | 51.3 | 64.3 | 0.112 |
| Bio-naïve (%) | 79.8 | 66.7 | 67.9 | 0.077 |
| MTX use (%) | 69.7 | 64 | 69.7 | 0.030 |
| MTX dose (mg/w) | 12 (8–16) | 12 (6–14) | 11 (6.3–16) | 0.120 |
| GC use (%) | 26.6 | 25.2 | 35.7 | 0.531 |
| GC dose (mg/d) | 5 (3–10) | 3.5 (2.5–5) | 5 (4.8–11) | 0.081 |
| CRP (mg/dL) | 1.6 (0.5–4.3) | 1.2 (0.2–3) | 4.8 (1.9–7.7) | <0.001 |
| ESR (mm/h) | 66 (39–87) | 62 (33–83) | 83 (61–101) | 0.001 |
| RF (IU/mL) | 70 (14–174) | 136 (50–283) | 110 (18–206) | 0.003 |
| ACPA positive (%) | 69.4 | 83.8 | 77.8 | 0.042 |
| TJC, 0–28 | 9 (5–13) | 8 (4–12) | 10 (6–14) | 0.396 |
| SJC, 0–28 | 7 (4–11) | 6 (4–10) | 10 (4–15) | 0.033 |
| PGA VAS, 0–100 (mm) | 58 (45–77) | 54 (42–75) | 74 (41–82) | 0.215 |
| EGA VAS, 0–100 (mm) | 50 (36–67) | 48 (33–60) | 46 (33–55) | 0.165 |
| CDAI | 29 (21–36) | 25 (19–32) | 30 (22–42) | 0.069 |
| HAQ-DI | 1.9 (1.3–2.5) | 1.9 (1.4–2.5) | 1.8 (1.4–2.6) | 0.852 |
| Pre-existing lung disease (%) | 47.7 | 43.2 | 42.9 | 0.775 |

Values are the median (interquartile range) unless indicated otherwise. Kruskal–Wallis and chi-square tests were used. yr = years; w = week; d = day; Stage = Steinbrocker’s stages; TNFi = tumor necrosis factor inhibitors; ABA = abatacept; TCZ = tocilizumab; Bio-naïve = biologics-naïve patients; MTX = methotrexate; GC = glucocorticoid; CRP = C-reactive protein; ESR = erythrocyte sedimentation rate; RF = rheumatoid factor; ACPA = anti-citrullinated peptide antibody; TJC = tender joint count; SJC = swollen joint count; PGA = patient global assessment visual analogue scale; EGA = evaluator global assessment visual analogue scale; CDAI = clinical disease activity index; HAQ-DI = health assessment questionnaire-disability index.

**Table S11.** Assessment of the generalized propensity score model after excluding patients who discontinued treatment because of remission

| AUC | TNFi  0.702 | | ABA  0.717 | | TCZ  0.730 | |
| --- | --- | --- | --- | --- | --- | --- |
|  | *p* | | *p* | | *p* | |
| IPTW | before | after | before | after | before | after |
| covariates |  |  |  |  |  |  |
| Age | <0.001 | 0.822 | <0.001 | 0.572 | 0.570 | 0.753 |
| Disease duration | <0.001 | 0.857 | <0.001 | 0.991 | 0.623 | 0.655 |
| Gender | 0.097 | 0.903 | 0.045 | 0.406 | 0.849 | 0.764 |
| History of bDMARD use | <0.001 | 0.789 | <0.001 | 0.298 | 0.054 | 0.805 |
| MTX dose | <0.001 | 0.923 | <0.001 | 0.844 | <0.001 | 0.702 |
| GC dose | <0.001 | 0.778 | 0.267 | 0.628 | <0.001 | 0.951 |
| TJC, 0–28 | 0.605 | 0.757 | 0.036 | 0.466 | 0.064 | 0.946 |
| SJC, 0–28 | 0.221 | 0.846 | <0.001 | 0.577 | 0.017 | 0.778 |
| PGA | 0.832 | 0.848 | 0.346 | 0.766 | 0.152 | 0.680 |
| EGA | 0.006 | 0.844 | <0.001 | 0.905 | 0.568 | 0.711 |
| HAQ-DI | 0.018 | 0.785 | 0.111 | 0.790 | 0.216 | 0.820 |
| CRP | 0.912 | 0.795 | <0.001 | 0.924 | <0.001 | 0.597 |
| ESR | 0.002 | 0.817 | 0.119 | 0.821 | <0.001 | 0.620 |
| RF | 0.339 | 0.934 | 0.067 | 0.961 | 0.353 | 0.997 |

Mann–Whitney U test, *t*-test, or tests for two independent proportions were used. AUC = area under the curve; IPTW = inverse probability of treatment weighting; TNFi = tumor necrosis factor inhibitors; ABA = abatacept; TCZ = tocilizumab; bDMARD = biological disease-modifying antirheumatic drug; MTX = methotrexate; GC = glucocorticoid; TJC = tender joint count; SJC = swollen joint count; PGA = patient global assessment visual analogue scale; EGA = evaluator global assessment visual analogue scale; HAQ-DI = health assessment questionnaire-disability index; CRP = C-reactive protein; ESR = erythrocyte sedimentation rate; RF = rheumatoid factor.

**Figure S1.** Flow chart of patient recruitment

RA = rheumatoid arthritis; bDMARDs = biological disease-modifying antirheumatic drugs.

**Figure S2.** Three-year retention rates of bDMARDs in non-adjusted data

Three-year retention rates of bDMARDs in all patients (A) and by age groups (B). yr = years; No. at risk = number at risk.

**Figure S3.** Three-year retention rates of bDMARDs in adjusted data using inverse probability of treatment weighting after excluding patients who discontinued treatment because of remission

Three-year retention rates of bDMARDs in all patients (A), in patients aged <65 years (B), patients aged 65–74 years (C), and patients aged ≥75 years (D). yr = years; TNFi = tumor necrosis factor inhibitors; ABA = abatacept; TCZ = tocilizumab.

**Figure S4.** Changes in CDAI in non-adjusted data

Changes in CDAI in all patients (A), patients aged <65 years (B), patients aged 65–74 years (C), and patients aged ≥75 years (D). Last observation was carried forward to assess changes in CDAI. Data are presented as mean ± 95% confidence interval (error bars). CDAI = clinical disease activity index; yr = years; TNFi = tumor necrosis factor inhibitors; ABA = abatacept; TCZ = tocilizumab.
